# Using PrISMa to reveal the interactome of the human claudins family

**DOI:** 10.1016/j.xpro.2023.102549

**Published:** 2023-09-26

**Authors:** Daniel Perez-Hernandez, Lorena Suarez-Artiles, Mattson S.O. Jones, Gunnar Dittmar

**Affiliations:** 1 Department of Infection and Immunity, Luxembourg Institute of Health, 1A Rue Thomas Edison, 1445 Strassen, Luxembourg; 2 Department of Life Sciences and Medicine, University of Luxembourg, 2 avenue de l'Université, Campus Belval, 4365 Esch-sur-Alzette, Luxembourg; 3 Max Delbrück Center for Molecular Medicine (MDC), Robert-Rössle Str. 10, 13125 Berlin, Germany

**Keywords:** High-throughput Screening, Protein Biochemistry, Proteomics, Mass Spectrometry

## Abstract

Here, we provide a protocol for the systematic screening of protein-protein interactions mediated by short linear motifs using the Protein Interaction Screen on a peptide Matrix (PrISMa) technique. We describe how to pull down interacting proteins in a parallelized manner and identify them by mass spectrometry. Finally, we describe a bioinformatic workflow necessary to identify highly probable interaction partners in the large-scale dataset. We describe the application of this method for the transient interactome of the claudin protein family.

For complete details on the use and execution of this protocol, please refer to Suarez-Artiles et al.^1^

## Highlights

• Identification protocol for SLiM-based protein interaction using a tiled peptide matrix
• Detailed protocol for the design and analysis of a PrISMa experiment
• R package for the analysis of PrISMa data

## Abstract (editor highlights)

Publisher’s note: Undertaking any experimental protocol requires adherence to local institutional guidelines for laboratory safety and ethics.

## Graphical abstract

**Figure:**
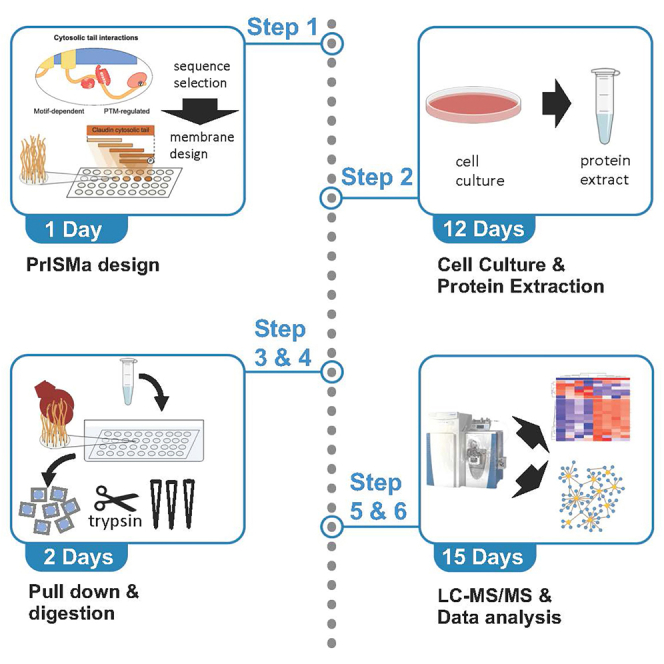

## Teaser

Here, we provide a protocol for the systematic screening of protein-protein interactions mediated by short linear motifs using the **Pr**otein **I**nteraction **S**creen on a peptide **Ma**trix technique. We describe how to pull down interacting proteins in a parallelized manner and identify them by mass spectrometry. Finally, we describe a bioinformatic workflow necessary to identify highly probable interaction partners in the large-scale dataset. We describe the application of this method for the transient interactome of the claudin protein family.

## Before you begin

Identifying protein-protein interactions can be difficult and mapping these interactions on the amino acids sequence of a target protein can be very laborious including the construction of many deletion constructs to narrow down the binding site. The PrISMa technique provides an alternative approach which is based on the use of synthetic peptides. The high concentration of the synthetic peptides on a peptide matrix can stabilize weaker interactions so they can be identified in the mass spectrometric detection.^1^^,^^2^^,^^3^^,^^4^^,^^5^ This protocol describes the PrISMa approach as a screening method to target interactions mediated by short linear motifs (SLiMs) in high-throughput.^5^^,^^6^ Since the peptide matrix is chemically synthesized, it allows the inclusion of modified amino acids, like amino acids carrying post-translational modifications or the inclusion of amino acid exchanges caused by mutations.

Here we describe the application of the method for the screening for protein interaction partners of the C-terminal tail of the human claudin family. Claudins are small transmembrane proteins and essential components of the tight junctions, which maintain the structure and permeability of endothelial cell membranes and form intercellular channels for small molecules.^7^^,^^8^

Here, we use nuclear-depleted protein extracts from MDCK-C7 cells to map interactions across linear C-terminal sequences from the human claudin protein family. The MDCK-C7 cells are broadly used for cell junction studies. This method matches the purpose of studying the all-in-once interaction profile in all human claudins. Nevertheless, this method can be also applied to nuclear or protein extracts from other cell types.^3^^,^^4^^,^^5^

**Figure 1:**
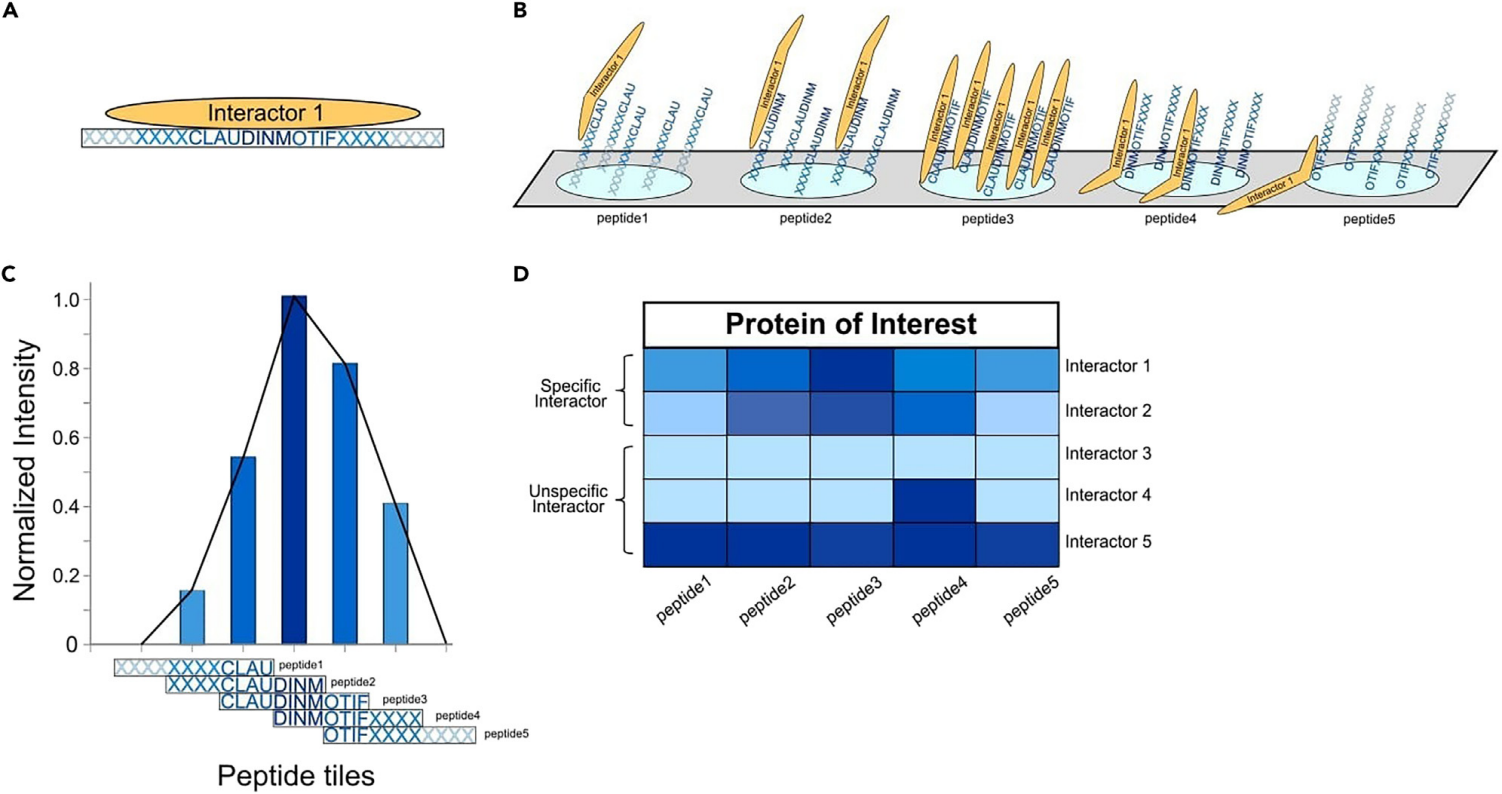
An illustrative example of positive and negative interactors in a PRISMA screen (A) An illustration of positive binding between a SLiM motif and an interaction partner. (B) Tiling peptide motifs on a PrISMa membrane allow gradual binding of interactors to specific motifs of the protein of interest. (C) Example of a positive interactor showing a gradual intensity between the PRISMA peptide tiles and the interactor. (D) Heatmap of an expected intensity profile of specific and unspecific interactors binding to tiled peptides. Specific interactors span multiple peptides and have a peak and gradual intensities, while unspecific interactors often bind a single peptide or multiple peptides with high intensities.

The protocol is divided into four steps (Graphical Abstract). First, we design the peptide screen of the claudin C-terminal region with post-translational modifications (PTMs) or mutations of interest. However, this can be applied to any protein or region of interest with high content in intrinsically disordered structure.^6^ Peptides should be around 13–15 amino acids long and tiled by 3–5 amino acids to the previous peptide (Figure 1). PTMs and/or mutations are included as separate entries.^6^ After designing the screen matrix, the chosen peptides are synthesized by Fmoc (Fluorenylmetyloxicarbonyl protecting group) chemistry in solid-phase peptide synthesis (SPOT)^9^^,^^10^ onto a PEG-cellulose membrane.^11^ In the second step, we isolate the protein lysate from MDCK-C7 cells needed for the PrISMa pull-down. Third, we incubate the MDCK-C7 protein extract on the PrISMa peptide membrane and pull down interactors. After subsequent washing to remove unspecific interactions, the spots are excised, denatured, and digested. Finally, we analyze the interactions by LC-MS/MS and filter the identified proteins and protein complexes that bind to specific regions based upon binding intensity in neighboring peptide tiling sites, either by sequence or by PTMs.^4^^,^^5^

## Key resources table

**Table:** 

REAGENT or RESOURCE	SOURCE	IDENTIFIER
**Chemicals, peptides, and recombinant proteins**		

Acetonitrile (ACN, MeCN, LC-MS grade)	Honeywell Riedel-de-Haen	Cat#34967
Ammonium bicarbonate (ABC)	Roth	Cat#T871.2
cOmplete Mini	Roche	Cat#11836153001
n-Dodecyl-beta-maltoside (DDM)	Bio Vision	Cat#2036-1
Dithiotreitol (DTT)	Sigma	Cat#D0632
Formic acid (FA)	Merck-Millipore	Cat#1.002640100
HEPES	Serva	Cat#25245.04
Iodoacetamide (IAA)	Sigma	Cat#I1149
KCl	Sigma	Cat#P9541
Methanol (MeOH, LC-MS grade)	Honeywell Riedel-de-Haen	Cat#34966
MgCl_2_	Ambion	AM9530
Modified trypsin, sequencing grade	Promega	Cat#V5113
NaCl	Roth	Cat#3957.1
Phosphate-buffer saline (PBS)	Gibco	Cat#14190-094
Penicillin-streptomycin solution 100×	Sigma	TMS-AB2-C
Protein Interaction Screen on Peptide Matrix (PRISMA)	JPT Technologies	PRISMA
Sep-Pak C18 96-well plates	Waters	SKU: 186003966
Serum-free DMEM	Gibco	Cat#41965062
STAGE tips : 3M C18 Empore disks	CDS Analytical	Cat#2215
Trifluoroacetic acid (TFA)	Merck/Millipore	Cat#1.08262.0100
Thiourea	Roth	HN37.2
Tris-HCl	Sigma	Cat#T4661
TritonX-100	Sigma	Cat#T8532
Trypsin/EDTA solution	Lonza	Cat#CC-5012
Urea	Sigma	Cat#U0631
Yeast tRNA	Invitrogen	AM7119

**Critical commercial assays**		

Bradford protein assay	Bio-Rad	Cat#5000006
Pierce BCA Protein Assay Kit	Thermo Fisher Scientific	Cat#23225

**Deposited data**		

Mass spectrometry proteomics data	ProteomeXchange	PRIDE: PXD031094
Proteomics databases for the claudin tails and the dog proteome	Mendeley Data	Dittmar, Gunnar; Suarez Artiles, Lorena (2023), “Reference proteomics databases for claudin prisma analysis”, Mendeley Data: https://doi.org/10.17632/ptxs8ctgb7.1 (V1)

**Experimental models: Cell lines**		

MDCK-C7 (canis lupus familiaris)		RRID: CVCL_0423

**Software and algorithms**		

MaxQuant version 1.5.2.8	Max-Plank Institute of Biochemistry	https://maxquant.org/
R version 3.5.0		https://cran.r-project.org/
RStudio version 1.0.143		https://www.rstudio.com/
ProTIGY Shiny app	Broad Institute	https://github.com/broadinstitute/protigy
prismaR package version 0.3.5	LIH	https://git.lih.lu/dittmarlab/prismaR
DAVID functional annotation tool	Huang et al.^12^; Sherman^13^	https://david.ncifcrf.gov
Metascape	Zhou et al.^14^	https://metascape.org/

**Other**		

nanoEASE M/Z peptideBEH C18, 1.7 μm, 100 μm ID, 100 mm analytical column	Waters	Cat#186008796
nanoEase M/Z symmetry C18, 5 μm, 180 μm ID, 20 mm trap column	Waters	Cat#186008821
Centrifuge adapters	GlyGen	Cat#5010-21514
Dionex Ultimate 3000 RSLCnano	Thermo Fisher Scientific	https://www.thermofisher.com/hplc-uhplc-systems/
Q-Exactive HF mass spectrometer	Thermo Fisher Scientific	https://assets.thermofisher.com/BR-64052-LC-MS-Q-Exactive-HF-Orbitrap
ReproSil-Pur C18 3 μm beads	Dr. Maisch	Cat#271118
23G needle	Fisher Scientific	Cat#123099179
0.2 mL Skirted 96-well Robotic Plate	Thermo Scientific	AB-1300

## Materials and equipment

**Table:** Mass spectrometer parameters: Q Exactive HF

General	
Runtime	0–9.8 min
Polarity	Positive
Default Charge State	2
**Full MS**	
Microscans	1
Resolution	60000
AGC target	3e6
Maximum IT	100 ms
Number of scan ranges	1
Scan range	375–1500 m/z
Spectrum data type	Profile
**dd-MS****^2^**	
Microscans	1
Resolution	15000
AGC target	1e5
Maximum IT	90 ms
Loop count	7
MSX count	1
Isolation Window	1.2 m/z
Isolation offset	0.0 m/z
Fixed first mass	30.0 m/z
NCE	28
Spectrum data type	centroid
**dd Settings**	
Maximum AGC target	4.50e3
Charge exclusion	Unassigned, 1, 6–8, >8
Exclude isotopes	On
Dynamic exclusion	15.0 s

**Table:** Nano High Performance Liquid Chromatography (HPLC) parameters: Dionex Ultimate 3000 RSLCnano

Time (min)	Flow (μL/min)	% Mobile phase B
0	0.300	2
2	0.300	10
8	0.300	40
8.1	0.300	90
9.8	0.300	90

**Table:** Maxquant version 1.5.2.8 parameters

Parameter	Setting
Reference Species	*Canis lupus familiaris* (UniProt 2018_08)
Reference Database	UniProt (2018_02) Claudin homo sapiens Database (database is available at https://doi.org/10.17632/ptxs8ctgb7.1)
**Settings**	Default
Variable modification	Methionine oxidation, N-acetyl at N-term protein and deamidation
Fixed modification	Cysteine carbamidomethylation
Quantitation	Label-Free Quantification (Fast LFQ)
Match Between Runs	Enabled

**Table:** Hypotonic Buffer A

Reagent	Final concentration	Amount
HEPES (1 M; pH 8)	5 mM	250 μL
KCl (1 M)	5 mM	250 μL
MgCl_2_ (100 mM)	0.75 mM	375 μL
cOmplete mini protease inhibitor cocktail		1 tablet
∗DTT (100 mM)	1 mM	500 μL
ddH_2_0	N/A	48.625 mL
**Total**	**N/A**	**50 mL**

∗add fresh before use.

∗∗only add prior to passing proteins through a syringe.

**Table:** Hypotonic Buffer B

Reagent	Final concentration	Amount
HEPES (1 M; pH 8)	5 mM	250 μL
KCl (1 M)	5 mM	250 μL
MgCl_2_ (100 mM)	0.75 mM	375 μL
DDM (100%)	0.5%	250 μL
∗DTT (100 mM)	1 mM	500 μL
ddH_2_0	N/A	48.375 mL
**Total**	**N/A**	**50 mL**

**Table:** Denaturation Buffer

Reagent	Final concentration	Amount
Urea (powder)	6 M	0.36 g
Thiourea (powder)	2 M	0.16 g
HEPES (1 M; pH 8)	20 mM	20 μL
ddH_2_0	N/A	1 mL
**Total**	**N/A**	**1 mL**

• **ABC buffer (50 mM ammonium bicarbonate buffer pH 8.5)**: 3.95 g of Ammonium bicarbonate in 1 L ddH_2_0. Adjust pH with NaOH or HCl.
• **DTT Buffer**: 100 mM DTT in ABC buffer (0.154 g of DTT in 10 mL of ABC buffer).
• **IAA Buffer**: 100 mM IAA in ABC buffer (0.184 g of IAA in 10 mL of ABC buffer). Prepare fresh before use in dark conditions.
• **Digestion Buffer**: resuspend each 20 μg vial of modified trypsin, sequencing grade in 80 μL 0.1% FA. Prepare fresh.
• **Elution Buffer A**: 40 mL ACN, 50 μL FA 100% in 9.950 mL ddH_2_O.
• **Elution Buffer B:** 25 mL ACN, 50 μL FA 100% in 24.950 mL ddH_2_O.

**CRITICAL:** Be careful when handling FA as it is corrosive and can burn the skin without gloves.

• **Mobile phase A** (HPLC): 0.1% FA in ddH_2_O.
• **Mobile phase B** (HPLC): 0.1% FA in 90% ACN in ddH_2_O.
• **MS Sample Buffer:** 500 μL 100% ACN + 25 μL 100% TFA in 49.475 mL ddH_2_O.

Store at RT for up to 12 weeks.

**CRITICAL:** Be careful when handling TFA as it is corrosive and can burn the skin without gloves.

• **DMEM + 10% FBS + 1**× **Penicillin/Streptomycin**: 50 mL FBS + 5 mL Penicillin/Streptomycin in 500 mL of DMEM medium.

Store at 4°C for up to 4 weeks.

## Step-by-step method details

### Design the PrISMa peptide membrane

**Timing: 3 h**

Here we design the peptides for the screening of the regions of interest. We will include the PTMs or mutations of interest as a separate entry on the screen. There is a company that commercialize those membranes in a cost-effective manner (JPT Peptide Technologies GmbH - JPT -). For our studies we utilized their membranes.

1. Select the region or the protein/proteins of interest.
2. Each selected region is designed based on structure and exposition to binding. Identify the amino acid sequence for the targeted region for utilization with the prismaR package (the amino acid sequence not as a fasta file). Here we use the Rad23 sequence file that is included in the prismaR package.
3. Create a meta description file using the *create_PTM_table()* command. The command creates a table which is needed to be filled in with the PTM information (The package contains an example dataset).
4. Read the meta data in using:

PTMs <- read_PTM_table()

5. Create the peptide list:

peptide_set <- construct_prisma_peptide_set(protein_sequence, PTM_table)

6. Order at least three replicates of the membrane from a peptide synthesis company that performs the Fmoc syntheses. Procedures to generate those membranes are described in.^9^^,^^15^

***Note:*** The overlapping peptides can be automatically designed using the prismaR R package (https://git.lih.lu/dittmarlab/prismaR). For a detailed description, see the documentation of the package. A high overlap of the peptides increases the resolution of the screen and makes the filtering of the results more effective, but also drastically increases the number of spots to analyze by LC-MS/MS, decreasing the time-cost effectiveness.

***Note:*** For peptides covering a modified site are all peptides spanning the modified site are also included as modified peptides besides the unmodified ones.

**CRITICAL:** The PrISMa screen works best for unfolded regions, like intrinsically disordered regions. Use a structural database to check if the selected region matches this criterion (PDB, UniProt).

***Note:*** In case a specific region contains several modifications, we recommend a maximum of three modifications per peptide. The number of control peptides added increases exponentially, hindering the analysis and the validation of the potential interactions.^5^

### Cell culture on MDCK-C7 cells and protein extraction

**Timing: 2 weeks**

Here we describe the generation of the nuclear-depleted protein extract from MDCK-C7 cells, following a modified version of a nuclear-cytoplasmic fractionation protocol.^16^

***Note:*** In this protocol, we use MDCK-C7 cells, which are well-established in the field of tight junction research. The cells can form tight junctions with a high frequency and thus provide a good source of tight junction proteins.^17^^,^^18^

7. Thaw MDCK-C7 cells in DMEM medium supplemented with 10% FBS and 1% Penicillin/Streptomycin and incubate in a 37°C and 5% CO_2_ humidified incubator.
8. Passage the cells at 80%–90% confluency.

***Note:*** 10 confluent 10 cm dishes are needed per PrISMa membrane. In this case, 166 spots were placed in a 5 cm × 4 cm membrane. Depending on the number of peptides to analyze the size of the membrane will differ, so the required material could be higher.

9. Wash the cells with 5 mL PBS twice.
10. Incubate with 1 mL trypsin/EDTA (0.25%/0.53 mM) solution for 1 h at 37°C.

**CRITICAL:** MDCK-C7 cells are well-known to be strongly attached to the culture dish. It is needed longer periods of time to detach the cells.

11. Collect detached cells in a 15 mL tube. See problem 1.
12. Centrifuge the cells at 600 g for 10 min at 4°C.
13. Remove the supernatant.
14. Resuspend cell pellet in 5 packed cell volumes (pcv) of Hypotonic Buffer A and incubate for 15 min on ice to allow cells to swell.

***Note:*** The buffer can be stored at 4°C for 4 weeks but If DTT has been added then only use fresh.

15. Centrifuge the cells at 600 g for 10 min at 4°C.
16. Remove the supernatant.
17. Resuspend the cell pellet in 5 cell pellet volumes of Hypotonic Buffer B.

***Note:*** The buffer can be stored at 4°C for 4 weeks, but if DTT has been added then only use fresh.

18. Pass cell suspension through a 23G needle 3 times to burst the cells without breaking the nuclei.
19. Incubate for 10 min at 4°C.
20. Centrifuge the lysate at 600 g for 5 min at 4°C to precipitate the nuclei.
21. Collect supernatant (post-nuclear supernatant) in a fresh 1.5 mL Eppendorf tube.
22. Collect 50 μL supernatant for protein concentration measurement, western blot analysis, and as a control for mass spectrometry analysis. See problem 2 and 3.
23. Snap-freeze the protein lysate at −80°C.

***Note:*** The cells should be at a low passage number (< 20).

***Note:*** If no interactions with membrane proteins are expected, we recommend an ultra-centrifugation step to clear membrane micelles from the lysate at 100,000 g for 30 min after step 12.

***Note:*** Protein quantification can be measured using a variety of assays following manufacturer instructions such as Pierce BCA Protein Assay (Thermo Fisher Scientific), Bradford Protein Assay (Bio-Rad), or other protein quantification methods.

**CRITICAL:** The protein concentration has to be maintained greater than 2 mg/mL. Lower concentrations will decrease the number of potential interactors drastically.^4^

**Pause point:** Samples can be used fresh. It is recommended to use protein lysates within 2 weeks of preparation. Samples can be stored at −20°C for 2 weeks; for longer periods they should be stored at −80°C.

### PrISMa pull-down

**Timing: 2.5 h**

Here, the protein extract is incubated with the peptide membrane to allow protein binding to the peptide spots.

24. Thaw cell lysate on ice.
25. Pre-condition the PrISMa membrane in a plastic container by submerging it in 5 mL Hypotonic Buffer A and incubating it on a rocking shaker for 45 min at room temperature (RT, at 22 motions per minute).
26. Wash the membrane 5 times with 5 mL Hypotonic Buffer A for 5 min each at RT on a rocking shaker.
27. Incubate the membranes in sealed bags with 2 mL MDCK-C7 cell extracts (3.5 mg/mL) for 20 min on ice in a rocking shaker.

***Note:*** For the membrane incubation, we recommend performing the binding in a sealed plastic bag, which fits the membrane to reduce the amount of protein extract needed for the experiment.

***Note:*** Multiple membranes should be incubated in separate bags.

***Note:*** We recommend performing the membrane binding assay in triplicates to obtain robust data for the downstream analysis.

28. Wash membranes 3 times with Hypotonic Buffer A for 5 min at RT on a rocking shaker at 22 motions per minute.
29. Let membranes dry on a clean glass surface for 60 min.
30. While waiting, fill Skirted 96-well Robotic Plates with 20 μL Denaturation Buffer per membrane spot (i.e., 166 peptide spots = 166 wells).

***Note:*** Samples should be analyzed at least in triplicates to further calculate the t-test comparison (Step 49).

***Note:*** The buffer has to be prepared fresh.

31. Cut membrane spots by cutting out each spot with a scalpel and transfer each spot with tweezers to a well containing Denaturation Buffer.

**Figure 2:**
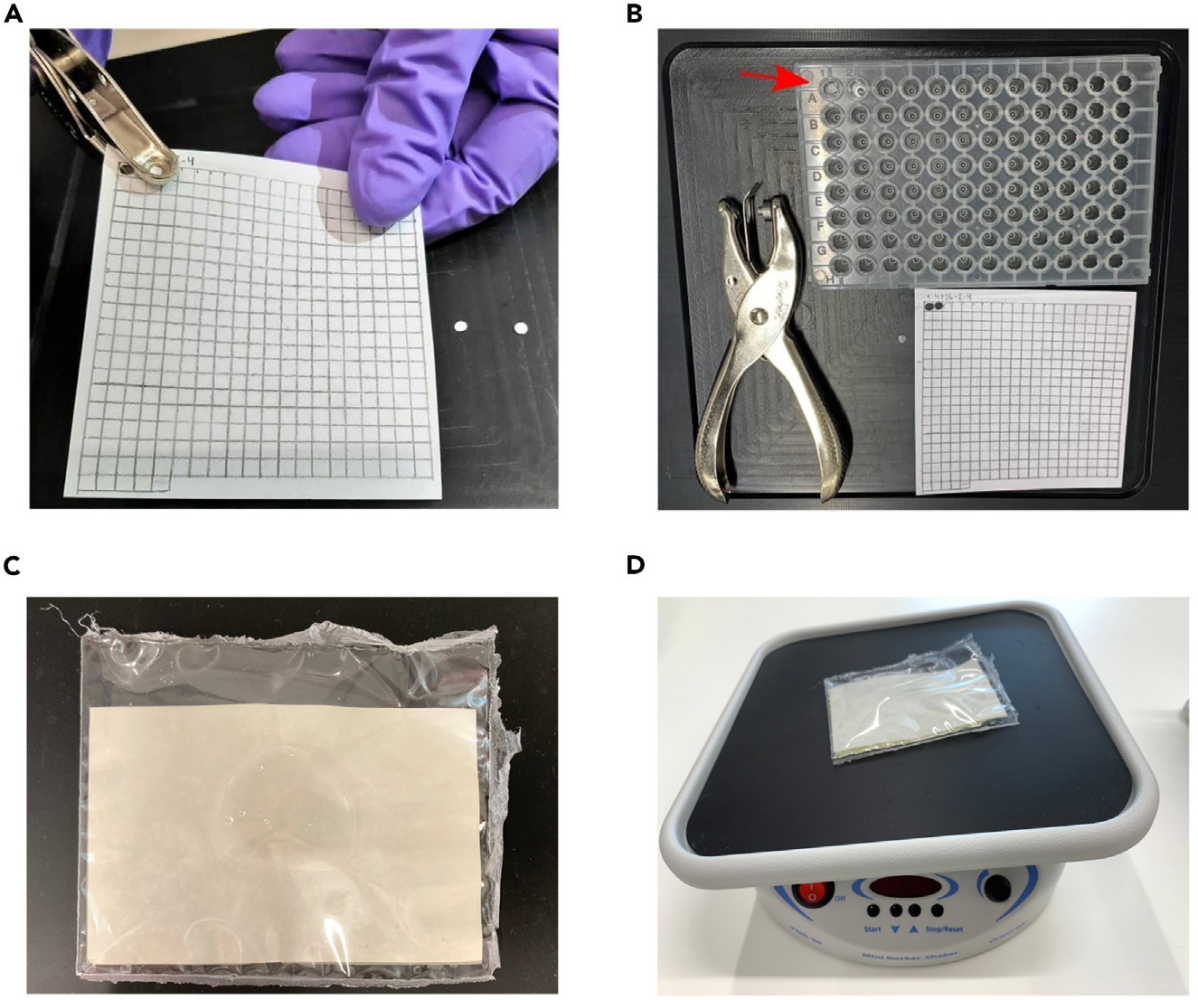
Extracting membrane tiles from the PrISMa membrane and incubation of the membrane (A) An example of a biopsy punch being used on a PrISMa membrane. Note the excised membrane tiles next to the membrane. (B) Tools used PrISMa study. The red arrow indicates excised membrane tiles placed into a conical 96-well plate containing buffer. (C) Sealed bag with the membrane and the cell extract. (D) Incubation of the sealed bag on a rocking shaker.

**CRITICAL:** The membrane should be cut when it’s dry to see the spots more clearly. The spots should be cut entirely as truncated spots will derive biased results.

***Note:*** A 25 mm biopsy punch can also be used to punch out spots from the membrane (Figures 2A and 2B).

**Pause point:** The Skirted 96-well Robotic Plate containing the excised peptide spots in Denaturation Buffer can be kept at −20°C for several weeks.

### In-solution protein digestion and peptide cleaning for LC-MS/MS analysis

**Timing: 1 h 30 min + overnight incubation**

Here we describe the steps to digest the protein extract bound to the peptide spots and desalting for LC/MS-MS analysis.

32. Add 1 μL DTT buffer to each well to the Skirted 96-well Robotic Plate containing proteins in Denaturation Buffer and incubate for 30 min at 37°C.
33. Add 3 μL IAA buffer to each well and incubate for 45 min at RT protected from light.

***Note:*** Reduction of disulfide bonds with DTT followed by alkylation with IAA is a standard procedure for mass spectrometry-based proteomics. The disulfide bonds are broken, and cysteine groups are carbamidomethylated (ΔM = 57.021), controlling the high reactivity of those groups, allowing the identification of cysteine containing peptides.

***Alternatives:*** Other reduction agents, like TCEP (Tris-(2-carboxyethyl)-phosphine), and alkylation reagents, such as Chloroacetamide can also be used.

34. Add 150 μL ABC buffer and add 2 μL of digestion buffer at 37°C overnight (ON).
35. Stop the digestion with 20 μL of 10% TFA and store the samples at −20°C.

**CRITICAL:** Check pH of the samples after adding TFA (pH < 2). Add more TFA in case the pH is > 2, otherwise there will be massive peptide losses in the peptide clean-up process.

**Pause point:** Samples can be stored at −20°C for several weeks.

### Peptide clean-up

**Timing: 1 h**

Here we described the desalting and cleaning of the digested peptides in a Sep-Pak C18 96-well plate.

36. Condition a Sep-Pak C18 96-well plate with 300 μL MeOH per well.
37. Centrifuge the plate at 700 g for 1 min at RT, removing the MeOH.
38. Add 300 μL elution Buffer A.
39. Centrifuge again at 700 g for 1 min at RT.
40. Perform two equilibration steps with 300 μL 0.1% FA with centrifugations at 700 g for 1 min.
41. Transfer the digested peptides to the plate.

**CRITICAL:** Check that the pH of the samples is < 3. Acidify by adding 1–2 μL of 10% FA if needed.

42. Wash and centrifuge at 700 g with 300 μL 0.1% FA 5 times, removing all the buffer.
43. Elute the peptides with 200 μL of elution Buffer B in a fresh Skirted 96-well Robotic Plate.
44. Dry peptides for 45 min - 1 h using a vacuum centrifuge until 5–10 μL remain in the well.

**Pause point:** Peptides can be stored at −20°C in the plate for several weeks.

45. Reconstitute dried peptides in 30 μL of MS Sample Buffer.

**CRITICAL:** After every centrifugation step, confirm that there is no liquid remaining on top of the wells. If liquid remains, centrifuge again at 1000 g for 3 min to remove it.

***Alternatives:*** Other methods can be used, like StageTips.^5^^,^^19^

### LC-coupled mass spectrometry

**Timing: 4 days**

Here we describe the analysis of the pull-downs by LC-MS/MS. In this protocol we used a Dionex Ultimate 3000 RSLCnano (Dionex) and Q Exactive HF (Thermo Fisher Scientific) using the Nano-electrospray ion source at 275°C. However, the HPLC-MS setup can be performed on different LC-MS/MS machines. Individual parameters should be adjusted considering identification depth and minimal LC gradient.

The PrISMa technique generates many samples for mass spectrometric analysis. In the case of the claudin samples, 798 peptide spots (166 spots run in triplicates) were analyzed.

46. Run samples on a nanoHLPC-coupled mass spectrometer in triplicates while running a blank between each sample.

***Note:*** We noticed a 5% carryover between samples and are encouraging the use of a blank to be run between each triplicate sample.

**CRITICAL:** Monitoring the blanks between samples will estimate the carryover. The levels should be maintained below 5%, otherwise the number of false positives hints will increase.

***Alternatives:*** Longer LC gradients can be used to identify more peptides. In our hands, this method is a good balance of time versus peptide identification.

### Data analysis using maxquant

**Timing: 1 week**

The recorded mass spectrometric data must be matched to peptides and proteins. For this, we use the MaxQuant software package (www.maxquant.org). MaxQuant demands significant computing resources. In our case, we used a 72-core computer to analyze the data. Using a less powerful computer will prolong the time needed for the analysis.

47. After collecting the raw data, run a MaxQuant software analysis using the parameters from the materials and equipment.

***Note:*** The proteomics reference databases for the human C-terminal sequences and the *canis lupus familiaris* proteome are available at Mendeley Data: https://doi.org/10.17632/ptxs8ctgb7.1.

***Note:*** In the MaxQuant analysis, two different databases were used. One for the sequences of the C-terminal regions of the human claudins and another database covering the *canis lupus familiaris* proteome. By checking for the peptide identifications mapped to the C-terminal regions database, peptides can be excluded that are derived from the peptide matrix, to avoid the identification of false claudin-claudin interactions (Filtering by reference species).

***Alternatives:*** Other search engine software could be used for data analysis (such as Proteome Discoverer, Mascot, Comet, etc.), but parameters and data analysis could differ from our workflow.

## Expected outcomes

This protocol provides a universal workflow to identify SLiM-mediated protein-protein or protein-complex interactions using tiling peptides. The interaction sites are mapped on the primary structure of the protein, allowing to pinpoint the site without the need for deletion mutant analysis. The number of interactions per peptide spot ranges from 350 – 700 proteins.^3^^,^^4^^,^^5^ In contrast to other peptide-pull-down studies, non-specifically binding proteins can be identified and removed by the intensity profile of the tiling peptides, allowing more confident results: only the proteins that fulfilled both binding and the intensity profile are considered as real interactions. The higher confidence in identifying new binding partners will minimize the number of candidates screened for further confirmation experiments and ensures a high removal of false positives.

## Quantification and statistical analysis

**Timing: 2 days – several weeks**

For the quantification and the statistical analysis, we recommend the use of the prismaR package (https://git.lih.lu/dittmarlab/prismaR). The package allows the fast analysis of the data and provides examples for the data analysis. In case of not using the prismaR package, you can follow the steps as outlined in the following paragraph using any data analysis software.

The analysis of the dataset is divided into several steps. First, the normalization step balances the high and low affinity interactions. The removal of background interactions filters the non-specific binding across all the spots. Next, the statistical analysis selects the potential candidates, and finally, the consecutive binding criterion defines the true positive interactors vs. the false positives.

Option 1: Analysis using the prismaR package

1. Read the MaxQuant data and cleanup the data (removing contaminants and reverse hits).

d <- read.delim("proteinGroups.txt")

d.c <- clean_proteingroups_data(d)

2. Create the meta data. Here we use the claudin23 datafile, which is included in the prismaR package.

create_prisma_ref(cldn23)

The command creates a file that can be opened with Excel to fill in the necessary information about the peptides, PTMs and replicates.

3. Read the filled in meta-data file.

reference_data <- read_reference(filename = "data")

4. If the prisma experiment contains replicates, these can automatically averaged.

ave_cldn23 <- average_binding_data(cldn23, reference_data)

***Optional:*** Peptide intensity can vary by orders of magnitude. Normalization of the data can be used to remove this and make downstream data analysis easier.

norm_cldn23 <- normalize_prisma_data(ave_cldn23)

5. Slim-based interactions should adhere to a specific binding pattern with the peptide with the highest binding signal being in the middle of the interaction pattern. To filter for interactions, which are obeying to this criteria use:

cons_cldn23 <- consecutive_binding(norm_cldn23)

***Note:*** In previous PrISMa studies the high background binding was detected for many proteins. In this case a filtering step with a 90% cut-off can be used to identify specific interactions despite the high background. In this case use out_cldn23 <- outlier_filtering(ave_cldn23, cutoff = 0.9) to remove the background.

Option 2: Steps for the analysis using other data analysis methods

6. For every claudin, select the LFQ intensities from the MaxQuant to calculate the intensity profiles for each identified protein group across all the monitored peptides.
7. The signal intensity for each protein group is normalized to a range of 0–1 across the matrix peptides by the following formula:

$$Normalized\;protein\;intensity=\frac{Intensity\;\left(spot\right)-\min\left(Intensity\right)}{\max\left(Intensity\right)-\min\left(Intensity\right)}$$

***Note:*** This step is important to recover proteins that are in low abundance but specifically interact with the spotted peptides and has been described in Dittmar et al., 2019.

***Note:*** Some proteins show some unspecific binding affinity, which can mask the specific interactions of this protein. To remove the background a filtering step as described in step 48 can be used.

8. Separate the interactions for every claudin in two different groups: all the unmodified peptides belong to group A, and the PTM peptides with their unmodified counterparts in group B. Statistical analysis of both groups is performed as follows:
  a. Group A: The unmodified peptides are analyzed separately for each claudin by moderated t-test pairwise comparisons with a significance cut-off of 5% False Discovery Rate (FDR) calculated by Benjamini-Hochberg correction.
  b. Group B: For the PTM analysis, we compare the modified peptides with their unmodified counterparts. We calculate the differences by moderated t-test pairwise comparison with a significance cut-off of 5% FDR calculated by Benjamini-Hochberg correction.

***Note:*** Spots with low or no intensity serve as controls for the binding sites.

9. The remaining interacting proteins are then tested for their binding behavior across several peptides (consecutive binding criteria, Figure 1C). Binding proteins should have the following intensity profile to be considered true positive interactors:

$$I_{n-2}\leq I_{n-1}<I_{n}>I_{n+1}\geq I_{n+2}$$

With n being the position of the peptide in the matrix.

The filtering can be done using an intensity profile which is applied across the row of binding intensities for each protein, filtering out every Intensity which is not obeying to the intensity profile. We can visualize the protein intensity profile by representing the LFQ values on a heatmap normalized by z-score (See problems 4, 5, and 6).

10. The analysis of the binding proteins can be extended using online software such as DAVID functional annotation tool (https://david.ncifcrf.gov) or Metascape (https://metascape.org/gp/index.html#/main/step1), correlating the different protein binders from one spot or across different spots, including Gene Ontology analysis or cross-correlation between proteins and protein-complexes.^4^

***Note:*** Besides the prismaR-package there are several free online software that can support the analysis, like Protigy or LFQ analyst.

***Note:*** For smaller datasets, the data can be visualized in a heatmap, and regions adhering to the consecutive binding criteria can be manually selected.

## Limitations

By design, the PrISMa technique can only identify interactions mediated by shorter interaction motifs. If interactions depend on folded structures or long amino acid stretches, these interactions will not be detected. If interactions based on the folded protein are expected, it is therefore recommended to complement the PrISMa protocol with Proximity labeling assays (BioID or APEX).

The PrISMa technique is well suited for the detection of transient or low-affinity interactions as the high concentration of peptides on the matrix stabilizes those interactions.^1^^,^^3^ The amount of material necessary for the analysis is higher than for AP-MS methods, although it avoids several IPs with truncated proteins for identifying the true binding site. Although PrISMa membranes can include PTMs in the screening, some larger PTMs, like ubiquitin, ubiquitin-like modifiers, or glycosylation, cannot be synthesized on the membrane. In this protocol, we reduced the time for LC-MS/MS analysis, comprising the RP gradient to 6 min. Still, large number of samples require larger time for the readout and analysis.

It is an important consideration to interpret the results that PrISMA is an *in vitro* screening method. The incubation of the synthesized peptides with the cell lysates might end up with protein binders that never meet under physiological conditions. Moreover, the 15-mer peptide structure could differ from the original protein. Hence, all the interactors must be confirmed using other methods.^6^ In previous studies, proximity ligations, co-immunoprecipitations, and Bio-ID assays successfully confirmed several interaction partners discovered by PrISMa.^3^^,^^5^

## Troubleshooting

### Problem 1

Cells don’t detach when treated with trypsin/EDTA solution.

### Potential solution 1

Avoid fully confluent plates. MDCK-C7 cells are very difficult to detach when they reach confluence.

### Potential solution 2

After removing the cell culture media, pre-incubate cells with the trypsin/EDTA solution for 5 min, remove, and add fresh trypsin/EDTA solution for the 1-h incubation time.

### Problem 2

Low protein concentration in the protein extract. Lysis conditions are too soft to burst cell membranes.

### Potential solution

Problems 2 and 3 are solved by adjusting the conditions to break the cells but not the nuclei. Before the last centrifugation step, take a 10 μL aliquot of the sample, add trypan blue, and inpsect the cells using a cell culture inverted microscope. Cells the number of intact cells should be below 1%, and nuclei should be intact.

Adjust lysis conditions accordingly by either omitting the syringe step or increasing the number of times it is used to cause mechanical disruption of the cell membrane.

### Problem 3

Western blot doesn’t show enrichment of nuclear proteins in the pelleted nuclei. Lysis conditions are too harsh to keep nuclei intact.

See the recommendations for problem 2.

### Problem 4

Low number of interactors (< 300 identified proteins).

### Potential solution 1

Alter Hypotonic buffer B detergent concentration. Decreasing the detergent concentration will increase the number of identifications.

### Potential solution 2

Decrease the number of washes.

### Potential solution 3

Increase protein concentration in the protein extract. The standard protein concentration on PrISMa is 2 mg/mL^4^. Higher concentrations will increase the number of proteins identified per spot.

### Potential solution 4

Optimization of the LC/MS-MS gradient. In this study, we used a 6 min effective gradient for LC/MS-MS analysis. Increasing the gradient to 20–30 min will boost the number of identifications, but it will also increase the time for sample acquisition.

### Problem 5

High number of interactors (high Background > 1200 proteins per spot).

### Potential solution 1

Reduce the concentration of protein extract to 2 mg/mL.

### Potential solution 2

Add two more washes in step 24.

### Problem 6

High number of False Interactors.

### Potential solution 1

Some highly abundant proteins, such as tubulins, histones, mitochondrial or ribosomal proteins from the protein extract, can mask the true interactors by saturation. Those interactions with high intensity across all peptides can be filtered computationally if they are highly abundant interactors across the membrane matrix.

### Potential solution 2

Increase the number of replicates to validate interactors.

## Resource availability

### Lead contact

Further information and requests for resources and reagents should be directed to and will be fulfilled by the lead contact, Gunnar Dittmar (Gunnar.Dittmar@lih.lu).

### Materials availability

MDCK-C7 cell lines are acquired from the European Collection of Authenticated Cell Cultures (ECACC).

## Data Availability

Mass spectrometry Proteomics data have been deposited at the ProteomeXchange Consortium via the PRIDE partner repository with the dataset identifier PRIDE: PXD031094. The amino acid sequences for the human claudins are available at Mendeley Data: https://doi.org/10.17632/ptxs8ctgb7.1. The PrismaR package is available from: https://git.lih.lu/dittmarlab/prismaR.
